# Lead Reconstruction Using Artificial Neural Networks for Ambulatory ECG Acquisition

**DOI:** 10.3390/s21165542

**Published:** 2021-08-18

**Authors:** Alejandro Grande-Fidalgo, Javier Calpe, Mónica Redón, Carlos Millán-Navarro, Emilio Soria-Olivas

**Affiliations:** 1 Analog Devices, Inc., 46980 Paterna, Spain; javier.calpe@analog.com (J.C.); monica.redon@analog.com (M.R.); carlos.navarro@analog.com (C.M.-N.); 2 IDAL, Intelligent Data Analysis Laboratory, Escuela Técnica Superior de Ingeniería, Universidad de Valencia, 46100 Burjassot, Spain; emilio.soria@uv.es

**Keywords:** cardiovascular diseases, electrocardiogram, ambulatory monitoring, lead reconstruction, artificial neural network, standard 12-lead system

## Abstract

One of the most powerful techniques to diagnose cardiovascular diseases is to analyze the electrocardiogram (ECG). To increase diagnostic sensitivity, the ECG might need to be acquired using an ambulatory system, as symptoms may occur during a patient’s daily life. In this paper, we propose using an ambulatory ECG (aECG) recording device with a low number of leads and then estimating the views that would have been obtained with a standard ECG location, reconstructing the complete Standard 12-Lead System, the most widely used system for diagnosis by cardiologists. Four approaches have been explored, including Linear Regression with ECG segmentation and Artificial Neural Networks (ANN). The best reconstruction algorithm is based on ANN, which reconstructs the actual ECG signal with high precision, as the results bring a high accuracy (RMS Error < 13 μV and CC > 99.7%) for the set of patients analyzed in this paper. This study supports the hypothesis that it is possible to reconstruct the Standard 12-Lead System using an aECG recording device with less leads.

## 1. Introduction

An electrocardiogram (ECG) is the registration of the electrical activity of the heart by recording the potential on the surface of the patient’s body. The most common way to record this information is the well-known Standard 12-Lead System [1], which requires ten defined electrode locations. This number of electrodes allows the system to have some redundancy and provides better projections to identify certain pathologies [2,3,4]. Nevertheless, the greater the number of electrodes, the greater the risk of problems related to the adhesion of an electrode or the deterioration of a wire and its connection to the recording device. This is also true for motion artifacts, which will be greater if there are more cables that may facilitate the appearance of this noise [5]. In addition, a larger number of electrodes increases the risk that the placement of electrodes may vary along the different records taken, which in turn increases the risk of ECG signal deviations [6].

To avoid all these complications, the reconstruction of missing ECG leads from a reduced set of leads is of increasing importance [7,8]. Most reconstruction techniques are used to reconstruct any unavailable leads from the redundant information inherent in the 12-Lead system [9]. Computerized algorithms have been developed to reconstruct the ECG of missing leads [10]. In the beginning, a set of reduced leads of the 12-Lead system were used, such as the II, V2 and V6 leads [11]. Dower later used a sub-system of Frank’s leads, the EASI Lead System, from which to extract the 12-lead standard system [12].

Reconstruction can be done by using either general or patient specific coefficients or models [13]. General methods are based on a unique transform matrix from which to extract the general coefficients, preventing the specific reconstruction for each patient, which is known to be inaccurate. A higher level of accuracy in the reconstruction is possible using specific reconstruction coefficients for each patient, reducing the deviation in the results for each patient. In practice, most current reconstruction techniques are used to reconstruct temporarily unavailable leads, for example, accidentally detached electrodes using the redundancy inherent to the Standard 12-Lead System. Based on the initial assumption that the electrical activity of the heart can be represented by a dipole model, or a cardiac vector, only three orthogonal leads should be necessary for the complete reconstruction of the electrical activity of the heart [9]. According to Willem Einthoven and Augustus Waller in 1903, this model was the accepted one. It is true that at this moment this is not the most accurate method to describe the model of the heart, as referred to so many times by the forward problem in cardiology [14], but the approximation is good enough to model the most basic reconstruction algorithms such as, for instance, the linear regression method [15].

To support the use of these methods, it has been shown that a reduced lead system may reproduce the information contained in the Standard 12-Lead System in controlled conditions [8]. This has been demonstrated by assessing the accurate reconstruction of the original 12-Lead ECG waveforms as well as comparing the diagnosis capabilities of both reconstructed and acquired ECG records. In conclusion, systems with a reduced number of leads may play an important role in ECG monitoring with a broader adoption in the out-of-hospital environment [16].

The aim of this study is to assess the feasibility to reconstruct the Standard 12-Lead System of a clinical ECG from a reduced number of leads acquired with an aECG device. Due to that reason, we were looking for a low cost, fast, low power consumption and relatively easy to retrofit system. These characteristics made us choose simpler machine learning techniques over more powerful techniques, but also more computationally expensive. To reconstruct the standard leads, different methods will be tested, from the commonly used Linear Regression method to the use of ANN. In all cases, the procedure involves personalizing the parameters for each patient. The remainder of the paper is organized as follows. Section 2 will describe the analyzed records and techniques applied. Section 3 will cover the results. Section 4 contains the discussion. Conclusions and suggestions for future work will follow.

### ECG Reconstruction Methods

To carry out the reconstruction of the ECG signal, many studies have been published that use a wide range of methods, as described below. Linear Regression reconstruction models are based on the cardiac vector reconstruction by using its projections in the captured leads [12]. This is carried out using Linear Regression adjustment, the oldest and most widely used method to perform the reconstruction. The goal is to minimize the sum of the squared errors to fit the data set. Thus, the voltage measured at an arbitrary point of the body, *V*, can be defined by Equation (1) as the projection of the cardiac vector, $\vec{H}$, with the corresponding vector generated by pointing to that point from the midpoint of the cardiac vector, $\vec{L}$ [17]:

(1) $$V=\vec{H}\cdot \vec{L}=aX+bY+cZ$$

where $\vec{H}=X\vec{i}+Y\vec{j}+Z\vec{k}$ and $\vec{L}=a\vec{i}+b\vec{j}+c\vec{k}$. Furthermore, *X*, *Y*, and *Z* coefficients can be replaced by any set of leads due to the linear condition of the cardiac model. In addition, *a*, *b*, and *c* are the transformation coefficients that can be modeled by a Least Squares Linear Regression model, providing the following solution for, as instance, three independent leads, $L_{1,2,3}$:

(2) $$\left[\begin{array}{c} a_{i}\\ b_{i}\\ c_{i} \end{array}\right]={\left[\begin{array}{ccccc} \sum L_{1}^{2} & \; & \sum L_{1}\cdot L_{2} & \; & \sum L_{1}\cdot L_{3}\\ \sum L_{1}\cdot L_{2} & \; & \sum L_{2}^{2} & \; & \sum L_{2}\cdot L_{3}\\ \sum L_{1}\cdot L_{3} & \; & \sum L_{2}\cdot L_{3} & \; & \sum L_{3}^{2} \end{array}\right]}^{-1}\cdot \left[\begin{array}{c} \sum V\cdot L_{1}\\ \sum V\cdot L_{2}\\ \sum V\cdot L_{3} \end{array}\right]$$

Which can be written as:

(3) $$NewLead=Coef1\cdot \vec{Lead1}+Coef2\cdot \vec{Lead2}+Coef3\cdot \vec{Lead3}$$

These coefficients can be applied to the entire signal, although the different waves that constitute the ECG can also be taken into account to select different coefficients, as suggested in past studies [18]. A common approach is to use two sets of coefficients, one for the reconstruction of most of the ECG and the other for the reconstruction of the much lower energy and highly clinically relevant P wave [9], as it is the reference for some pathology diagnostics, such as left ventricular interstitial fibrosis [19].

The EASI Lead System plays an important role in this field. Several works have proposed this system to carry out the reconstruction by means of general coefficients that can be tuned for each patient or application [20]. In this aspect, it has been demonstrated that a satisfactory reconstruction of the Standard 12-Leads using EASI ones can be performed, allowing its use in pathologies diagnosis [7,8,20,21,22,23].

Around the 1950s, new data processing systems based on a simplistic version of brain functioning emerged. These systems are called ANN and are one of the most widely used systems in the field of Machine Learning algorithms to date. A conventional ANN is based on a set of connected nodes called neurons that roughly mimic the functioning of a neuron in a real brain. These neurons gather information collected from other cells, process it, and then transmit another signal through synapses towards other neurons, thus allowing the processing and transfer of information [24]. In an ANN, these signals are actually numbers, which enter each neuron and are adjusted by a weight that is what learning regulates. In the neuron, they are computed by a non-linear function and a comparison with a threshold determines if the signal is propagated or not to the rest of the neurons connected to it [25].

Neurons that form the network are usually aggregated in layers. The first layer is known as the input layer, where data or signals are fed in. This information is processed by the hidden layer(s), and the final result comes from the output layer.

There are some cases where ECG reconstruction does not optimally perform with linear approximations. Non-linear methods, such as ANNs, may provide more accurate reconstructions in situations where linear methods fail [26]. In the field of ECG lead reconstruction, one of the procedures is to take some lead signals from the Standard 12-Lead System as input parameters and then use them to reconstruct other leads at the output of the ANN. They may improve the ECG leads reconstruction obtained with Linear Regression [16,18,25,26]. As the method proposed by [26], where their method based on ANN reconstruction is so robust that the differences between original ECG and the reconstructed ECG were due to electrode misplacement, and not provoked by the method itself.

There are several ways to increase the performance of these systems, for example, by incorporating them into ANN committees to improve the robustness and accuracy [16] or by applying genetic algorithms for the extraction of features from learning data [18,27].

Currently, several teams are addressing this challenge using the Deep Learning approach, which has gained popularity in recent years. To do so, they use complex models with a high number of parameters to carry out this task. From reconstruction using Long-Short Term Memory (LSTM) cells [28] to Convolutional Neural Networks (CNN) [29], passing through regression trees [30], these models are computationally demanding, and we decided to take a different approach by using simpler models that are able to perform the same task with the same level of accuracy.

## 2. Materials and Methods

### 2.1. ECG Recordings

**Figure 1:**
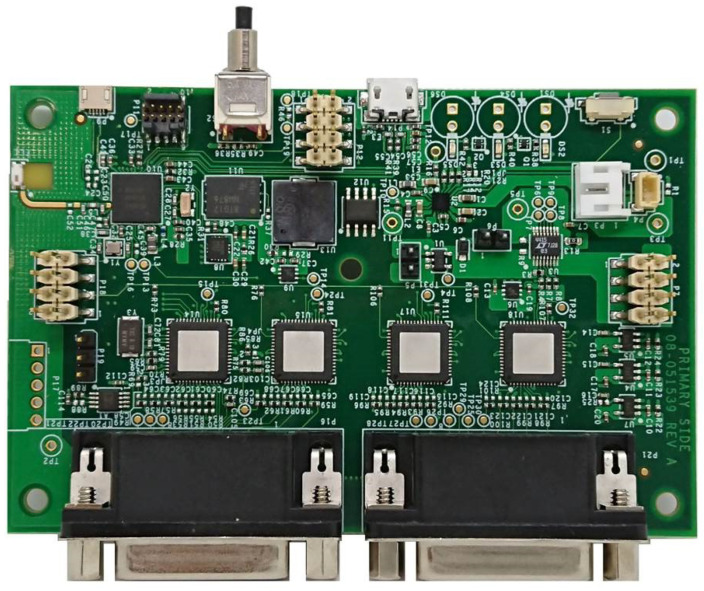
ADAS1000 based board for high quality ECG signal recording.

The ECG records required for the study have been acquired using a proprietary system shown in Figure 1 based on the ADAS1000 analog front end, from Analog Devices Inc. [31]. This system is a 19-channel synchronous acquisition system, sampling at 1kHz with a 16-bit resolution using four ADAS1000.

Electrocardiogram records lasting between 60 s and 120 s were recorded from five men and two women, aged from 23 to 54 years, with different morphologies. They have been obtained accomplishing GDPR regulation by ensuring full patient anonymization. One of them presented a right bundle branch block and another one had premature ventricular contractions. As previously mentioned, data from 19 channels were captured. Subsequently different subsets of those 19 channels were analyzed, e.g., the Standard 12-Lead System, the 3 EASI Leads, as well as different 3-leads combinations.

The ECG records were preprocessed to mitigate noise. Two 4th order Butterworth IIR bi-directional filters were applied. A low-pass filter with 150 Hz cut off frequency mitigates myoelectric noise and high-frequency interference, and a high-pass filter with a 0.67 Hz cut-off frequency reduces baseline wandering and offset.

### 2.2. Reconstruction Algorithms

**Figure 2:**
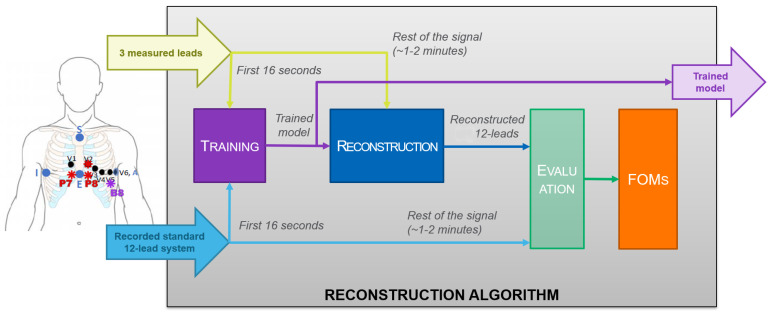
Reconstruction methodology.

As previously mentioned, to obtain the Standard 12-Lead System ECG, theoretically, at least three ECG leads are required [11]. This minimum use case of three leads is used in this work. Reconstruction was carried out as described in Figure 2. The reconstruction algorithm model was trained for a time that was empirically determined, and the rest of the record was processed with the obtained model. The goodness of the reconstruction was evaluated comparing the estimated signal with the actual one recorded at the standard leads position using the five Figures of Merit (FoM) that will be defined in Section 2.3.

Four strategies for reconstruction were implemented. Two are based on linear regressors and two are based on ANNs. Linear Regression by minimum squares reconstruction models are based on the cardiac vector reconstruction by using its projections in the captured leads. The goal is to model the cardiac vector representation on each lead from the Standard 12-Lead system by the Least Squares Linear Regression, as described in (2) and (3). This is a well-known approach, and it will be the control case from which to compare the rest of the algorithms and assess their efficacy.

**Figure 3:**
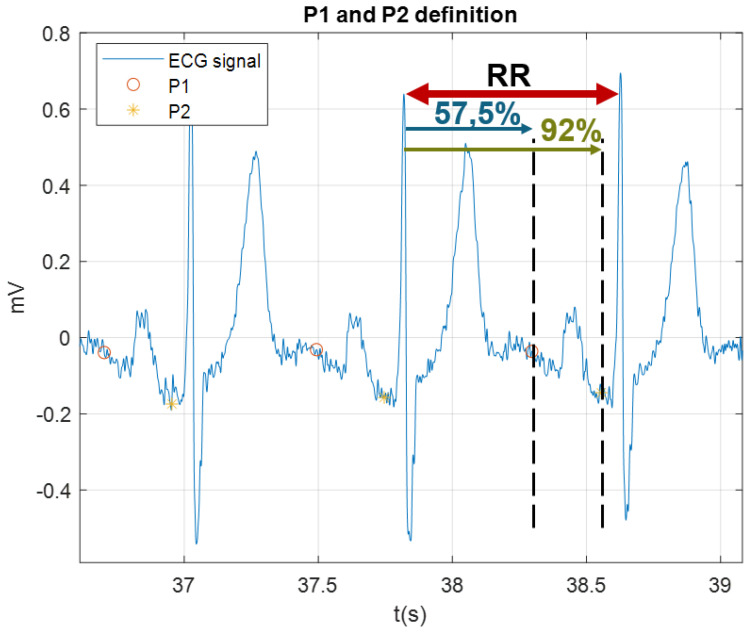
Methodology of P wave segmentation.

**Figure 4:**
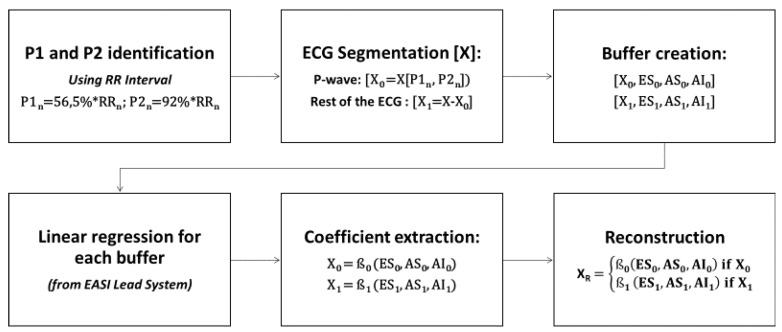
Block diagram of the P wave segmentation algorithm to reconstruct the ECG signal.

The least squares method tends to focus on the areas with higher energies, which is the QRS complex in the case of the ECG, and may ignore other waves with lower energies in the ECG signal such as the P-wave, which decrease its reconstruction performance. A variation is proposed that consists in dividing the ECG signal in two segments, separating the P wave from the rest of the beat. To generate the ECG segmentation, it is necessary to identify some fiducial points. In this case, since only the identification of the P wave was necessary, the delimitation of it was carried out from the detection of R peaks and the measurement of RR intervals. From this, the beginning and the end of the P wave, P1 and P2, respectively, are defined in terms of relative percentage of duration of the RR interval. The values empirically obtained were 57.5% of the RR interval for the beginning of the P wave and 92% of the duration of the RR interval for the end of the P wave, as shown in Figure 3. We generate two sets of buffers with the 3 original signals. In this case, derivations of the EASI: ES, AS and AI, and the signal to reconstruct, X The first buffer is labeled 0, and contains the P-wave fragment. The second buffer, marked as 1, contains the rest of the signal. Then, we obtain two different sets of coefficients from the three original signals from the EASI, β0 and β1, from both set of buffers, respectively. Finally, the two linear regressors with the coefficients are applied: one for the P wave segment, and a second one for the rest of the ECG signal, as indicated in Figure 4. These results will then be concatenated and compared with the original signal.

The other two approaches are based on ANN using a Multilayer Perceptron. To synthesize each of the 12 leads from the three acquired leads, a one hidden layer feed-forward ANN system is trained with the Levenberg–Marquardt algorithm [32]. The non-linear transfer function applied in the hidden layer is the hyperbolic tangent sigmoid function.

**Figure 5:**
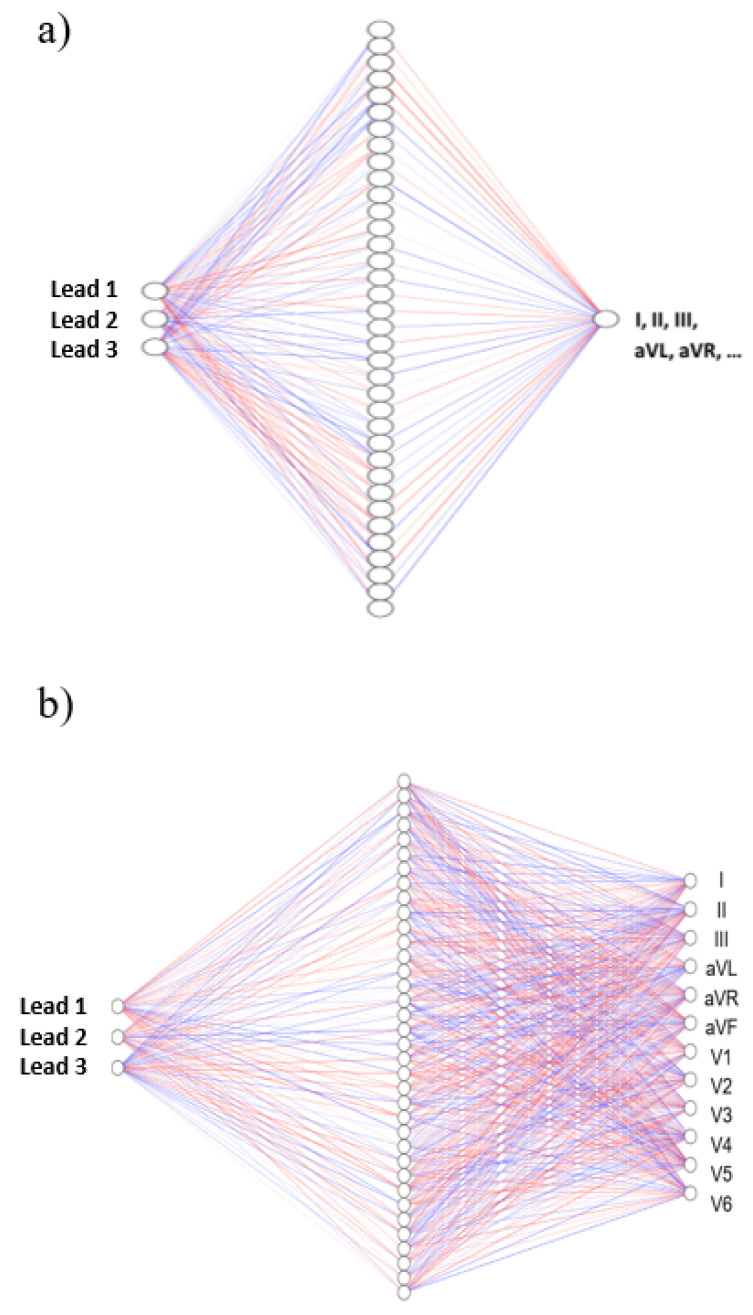
ANN to reconstruct ECG: (**a**) single lead of the Standard 12-Lead system and (**b**) all leads of the Standard 12-Lead System simultaneously.

Two different strategies are explored. One of them uses 12 ANNs (ANN/Lead), one per reconstructed lead (Figure 5a). Therefore, each ANN has a single neuron in the output layer. On the other one, a single ANN has 12 output neurons (All-Lead ANN), one per lead (Figure 5b). The same number of neurons in the single hidden layer was used for both architectures, which was decided empirically based on a sweep of different layer sizes, choosing a trade-off between the optimal result and a convenient computational expense. This was obtained empirically based on the results of the stability and accuracy for the most demanding of the two models, which is the reconstruction of the ANN with twelve outputs. In this way, we may fairly compare both architectures, as the single output ANNs are simpler and might require fewer neurons in the hidden layer.

### 2.3. Reconstruction Assessment

The choice of the FoMs to evaluate the accuracy of the reconstruction is not trivial. Choosing certain FoMs and not others will lead us to evaluate some characteristics and not others. In [33], they conduct a study on what should be a robust set of parameters to evaluate the fidelity of the ECG signals. Although this work was done for ECG compression, its principles are applicable to our field, as we also elaborate a kind of ECG decompression. According to the results and conclusions from [33], and our own analysis, five FoMs were selected to assess the reconstruction quality of the ECG signal:

Root Mean Square Error, *RMS* or $V_{RMS}$, is mathematically described by Equation (4), where $x_{n}$ is the original signal, ${\tilde{x}}_{n}$ is the reconstructed signal, and *n* is the index of each sample of the signal of length *N*. $V_{RMS}$ has the advantage that it keeps the original units of measure, millivolts (mV), although here it has been converted to microvolts (μV) to facilitate its understanding in future graphic representations [34].

(4) $$V_{RMS}\left(\mu V\right)=\sqrt{\frac{1}{N}\sum_{n=1}^{N}|{\tilde{x}}_{n}-x_{n}{|}^{2}}$$

Cross Correlation, *CC*, expressed as a percentage % is defined by Equation (5), where $\mu_{x_{n}}$ and $\mu_{{\tilde{x}}_{n}}$ are the global averages of the original and reconstructed signals, respectively, and $\sigma_{x_{n}}$ and $\sigma_{{\tilde{x}}_{n}}$ are the standard deviations of the original and reconstructed signals, respectively, in addition to the variables defined above. As mentioned before, many authors use this method, so the new results can be easily compared with theirs [34,35]. Nevertheless, the CC is not an accurate estimator of the reconstruction goodness, because it rests most of its value in the fitting of the isoelectric line and, in any case, to the high energy QRS complex. The rest of the ECG wave morphology does not substantially affect this parameter, which may be critical to the diagnosis of certain pathologies.

(5) $$CC\left(\%\right)=100\cdot \frac{1}{N-1}\sum_{n=1}^{N}\left(\frac{{\tilde{x}}_{n}-\mu_{{\tilde{x}}_{n}}}{\sigma_{{\tilde{x}}_{n}}}\right)\left(\frac{x_{n}-\mu_{x_{n}}}{\sigma_{x_{n}}}\right)$$

Maximum Amplitude Distance, or Maximum Amplitude Error, *MAD* or MAX, is mathematically described by Equation (6). It brings information about local distortion of the signal and is usually calculated separately for each cycle, while here it was measured for the complete recording. *MAD* also maintains the original units of measure, millivolts (mV), although here, as with RMS, it has been converted to microvolts (μV) to facilitate graphic representations. It is one of the most used similarity metrics [33,35].

(6) $$MAD\left(\mu V\right)=\max_{n}|{\tilde{x}}_{n}-x_{n}|,1⩽n⩽N$$

Sum of the Square of the Distances, *SSD*, is defined by Equation (7). It allows us to measure the accumulated error and gives an approximation about how the signals differ in their full length [35]. As it is the square of the differences, its measurement units are, in this case, square millivolts ($mV^{2}$), to simplify its interpretation with the rest of the parameters.

(7) $$SSD\left(mV^{2}\right)=\sum_{n=1}^{N}{\left({\tilde{x}}_{n}-x_{n}\right)}^{2}$$

Signal to Noise Ratio, *SNR*, takes noise as the difference between the original signal and the reconstructed signal, as described in Equation (8), where $\tilde{x}$ is the reconstructed signal and $\bar{x}$ the mean of the original signal. As SNR expresses signal levels, its measurement units are decibels (dB).

(8) $$SNR\left(\mathrm{dB}\right)=10\cdot \log_{10}\left(\frac{\sum_{n=1}^{N}{\left[x_{n}-{\tilde{x}}_{n}\right]}^{2}}{\sum_{n=1}^{N}{\left[x_{n}-{\bar{x}}_{n}\right]}^{2}}\right)$$

The five FoMs are obtained for each reconstruction method and electrode location. The evaluation of significance between the control method and the rest of the reconstruction methods will be done by means of the Wilcoxon rank sum test [36] for each FoM.

### 2.4. Leads Placement

**Figure 6:**
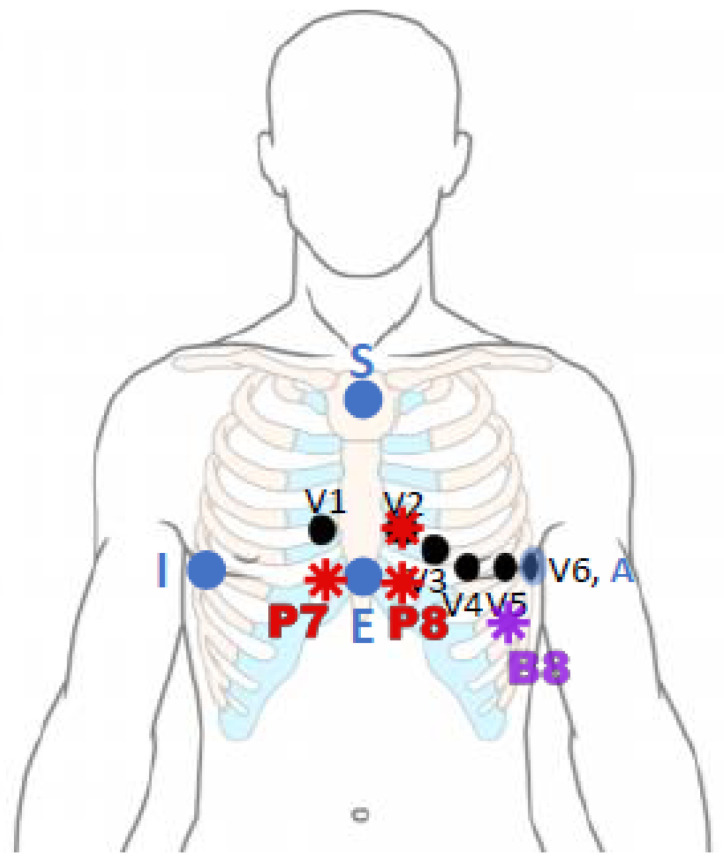
Additional electrodes location. In blue dots, the Dower location, and in red and purple, our proposed location. Red dots (V2, P7 and P8) are located in the patient’s chest, and the purple one (B8) is placed in the back. In black, the standard precordial leads are shown for reference.

To validate the technique, in addition to the ten 12-Lead electrode locations, we consider the Dower location as it is supported by prior publications [12], and we also propose additional locations that might result on electrodes positions that adapt better to both physiological and anatomical constrains, such as a voluminous breast, a scar in standard positions, etc. It involves four electrodes, three of them located on the chest. V2 is the same as in the Standard 12-Lead System, P7 is located in the fifth intercostal space just to the right of the sternum, P8 is located In the fifth intercostal space just to the left of the sternum, and B8, horizontally following the line that V5 and V6 form, opposite to V5. Thus, forming three leads: P8-P7, V2-B8 and P8-B8. See layout in Figure 6. These leads were selected for several reasons. They are closer together than those forming the EASI, which makes placement and wearability more comfortable. The formed leads form a set of quasi-orthogonal leads, in the same direction as the EASI does. Furthermore, those leads returned the best reconstruction results.

## 3. Results

The two linear regressors and both ANN methods are trained for every patient in order to achieve the best results. The size of the training set was empirically established, and is set to 16 s (16,000 samples). The remaining of the record, 104 s (104,000 samples), is used for the test. All results shown have been obtained from the test part of the rest of the records.

**Figure 7:**
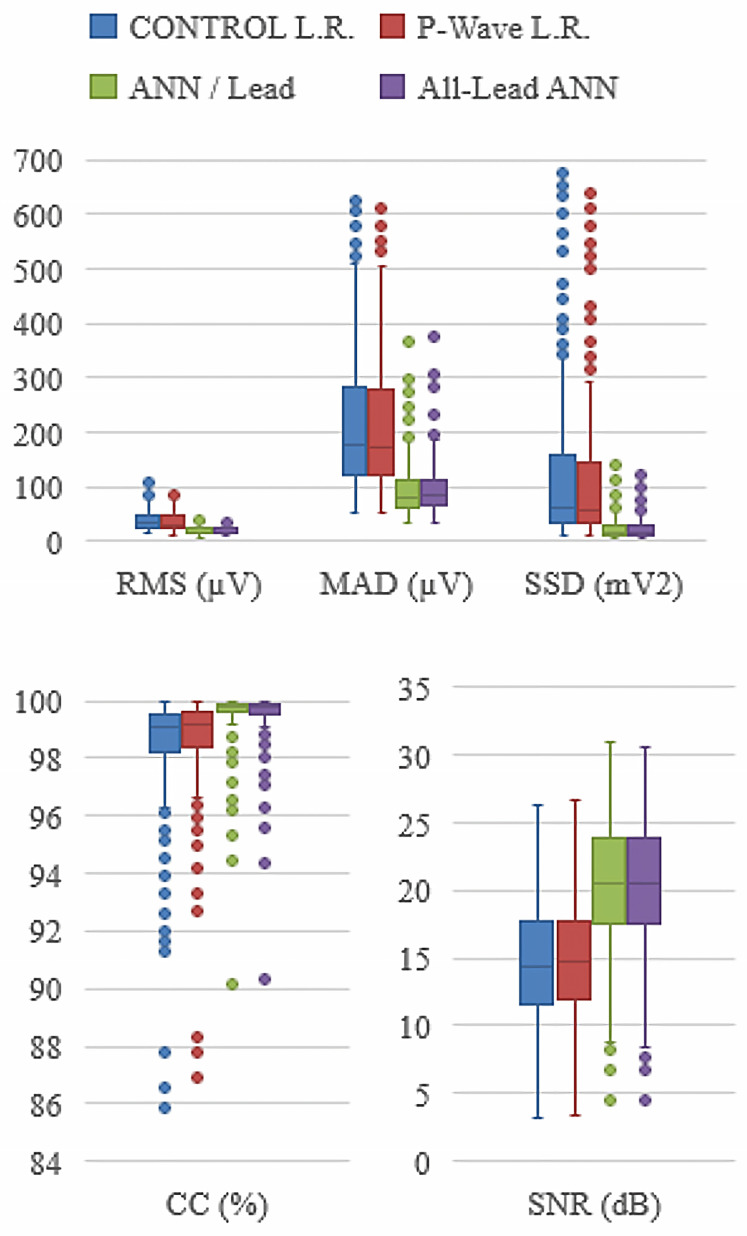
Boxplot graphical representation of the five FoMs for each of the four ECG reconstruction methods studied. The results for both Linear Regression methods are represented in blue and red for the simple Linear Regression method and the P-wave segmentation method, respectively. The ANN strategies are represented in green and purple for the ANN/lead and the unique ANN methods, respectively. The parameters have been divided into three graphs to facilitate their visualization and interpretation.

The results have been expressed in terms of median and interquartile ranges represented in the form of boxplots in Figure 7. The results for both Linear Regression methods are represented in blue and red for the simple Linear Regression method and the P-wave segmentation method, respectively. The ANN strategies are represented in green and purple for the ANN/lead and the unique ANN methods, respectively. Independence of each group of results has been studied for each FoM between the control method (simple Linear Regression) and the other three proposed reconstruction methods, as shown in Table 1. The differences of all the FoMs in both methods involving ANN with respect to the control are highly significant. On the contrary, the P wave segmentation method does not show significant differences for any of the five FoMs. No significant difference was found between the two ANN-based algorithms by means of the Wilcoxon test. Figure 7 shows the FoMs for each method to reconstruct the Standard 12-Lead System from the three leads of the Dower location.

The results for both ANN strategies are significantly better than the Linear Regression methods for the five FoM analyzed. There are no meaningful differences between the original signals and the reconstructed ones by both strategies based on ANNs. As for the execution time, a single network with 12 outputs is slower to converge than the 12 single output networks. For that reason, this is the preferred implementation, and it is very likely that these networks would require less neurons in the hidden layer, although this requires a more thorough validation and it is beyond the purpose of this paper.

**Figure 8:**
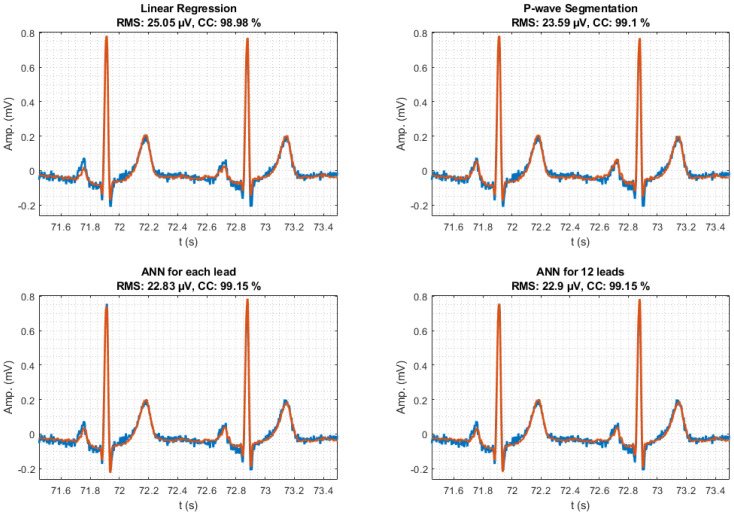
Reconstruction by the described methods of an ECG fragment of lead II of one of the patients. To perform the reconstruction, EASI leads were chosen. Two of the five FoMs, RMS error and CC, are indicated. In blue, the original signal and, superimposed in orange, the reconstructed signal.

An example of the reconstruction of an ECG fragment of lead II from a random patient has been shown in Figure 8. This choice was randomly determined to ensure no bias in the quality of the reconstruction, either by a healthy or pathologic patient or by other biases such as signal quality due to skin type. It can be seen that the reconstruction is acceptable in all cases, being, as the general results in Figure 7 show, better in the case of reconstruction by ANN than by both linear approaches. An underestimation of the amplitude can be appreciated in Figure 8, where these second type of regressors in the reconstruction of the P wave, as well as the peaks of the QRS complex, especially in the Q and S waves.

**Figure 9:**
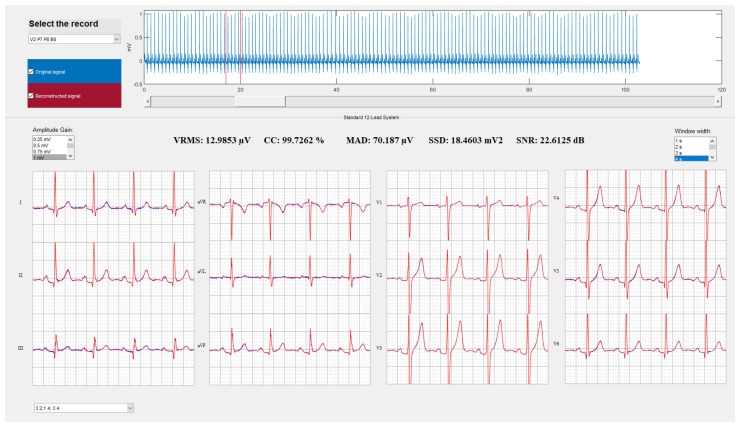
Reconstruction of the Standard 12-Lead System applying the 12-output ANN-based algorithm for the set of electrodes and leads chosen as the best option. First position was obtained by electrodes V2, P7, P8 and B8; arranged in leads P8-P7, V2-B8 and P8-B8. The original 12 leads are represented in blue, and the 12 reconstructed leads are superimposed in red.

Table 2 shows the results obtained in Figure 9, which is the reconstruction using the alternative position proposed by the authors, which is leads P8-P7, V2-B8 and P8-B8, as indicated in Figure 6.

## 4. Discussion

The reconstructions carried out by the algorithms based on both ANN approaches are visually perfect, there are no differences between the original signals and the reconstructed ones, as shown in Figure 9.

Table 1 shows the comparison in terms of the significance between the sets of results obtained in reconstruction using the methods cited. We did not obtain significant values of independence between the FoMs obtained from the simple Linear Regression and the Linear Regression with two sets of coefficients, one of them for the P-wave. In contrast, both groups corresponding to the ANN reconstruction showed a very high significance in their independence from the control group, the Linear Regression. For these sets, the evaluation values of the RMS, MAD and SSD reconstruction adjustment fell sharply, and the CC and SNR increased. Our results are supported by the results of other publications on the topic [16,26,37], which confirm that the best algorithm for reconstructing the electrocardiographic leads of the Standard 12-Lead System is the artificial neural networks reconstruction algorithm.

Compared with other works, our proposed location for the reconstruction improves the reconstruction of the Standard 12-Lead System when compared to others that use electrodes arranged in a pseudo-arbitrary way on the patient’s chest. Ref. [37] obtains, with their best configuration, a mean value of 97.9% for the CC and a mean value of 27.4 μV for the RMS Error. Ref. [30] obtains a mean value of 98.7% for the CC. Our proposed location obtains a mean value of 99.73% for the CC and a mean value of 12.99 μV for the RMS Error.

**Figure 10:**
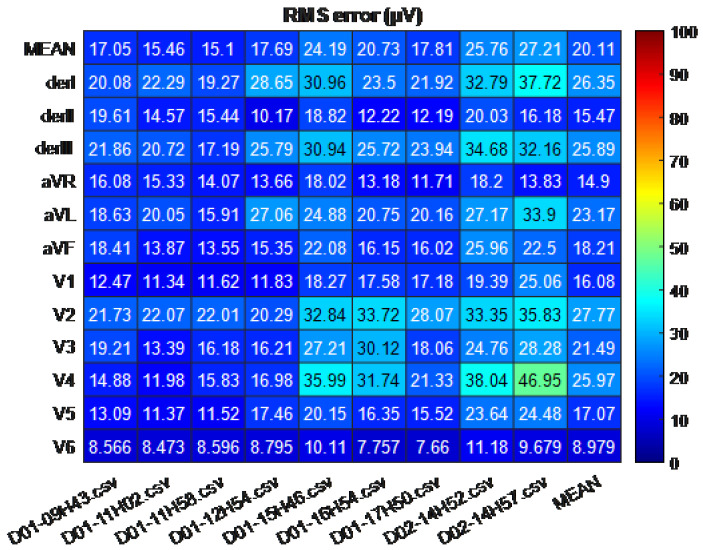
Results for one of the FoMs (the RMS error) where the reconstruction is evaluated over several days at different time instants. To carry out this reconstruction, the algorithm (ANN for each lead) was only trained with the first 16 s of the first record, and the model performed the reconstruction of the rest of the records, without any feedback. The x-axis shows the records, identified with the day D and the time xxHxx at which they were taken, and the y-axis shows the standard leads, as well as their mean RMS Error value for each record.

The robustness of the reconstruction algorithm was validated by acquiring records from the same individual at different times and days and the reconstruction was carried out without retraining. No significant degradation in performance was noticed as shown in Figure 10.

We also verified the quality of the reconstruction for different positions, mostly fowler and supine, of the patient while at rest. No errors were observed even if the patients modified their position from training to test. In addition, records obtained in the presence of moderate physical activity showed no worsening in their reconstruction.

One of the problems with the choice of these FoMs is that if the original signal with which to compare the reconstruction presents noisy artifacts, the FoMs will worsen, since the reconstructed signal tends to be cleaner and not matches the original, as seen in Figure 8. In this example, the reconstructed signal, in orange, is smoother than the original, the blue one. This smoothing is due to the absence of noise that the reconstructed signal has, which is caused by, among other reasons, the use of several leads to reconstruct, which reduces the noise of the resulting signal. This may be misleading as the actual ECG signal does not contain those disturbances, as it is noise added to the signal, so FoM should always be critically analyzed. Furthermore, when we reconstructed a certain lead under moderate exercise we might observe that the actual signal had poorer quality than the reconstructed one as was affected by electromyographic noise.

The main limitation of this study is the lack of records in databases with sufficient time to carry out the training and testing process. We need databases that contain many channels, not only those of the Standard 12-Lead System, but others with which to train these additional configurations. That is why the records must be taken by ourselves, which means a delay in the ability to get new patients.

## 5. Conclusions

This work has demonstrated the feasibility of an aECG system that might continuously and non-intrusively acquire ECG records from a small set of electrodes and reconstruct the signals of the Standard 12-Lead System, the most extended method in clinical procedures, after a short training, specific for each patient. We have explored different strategies to reconstruct the ECG, the best being reconstruction by ANN. In addition, an optimal position has been proposed.

This work makes a more exhaustive analysis of the quality of the reconstruction than the vast majority of studies, which only focus on the RMS error and the CC. These two FoMs tend to undervalue the quality of the areas of the ECG with less energy. We emphasized the reconstruction of less energetic areas of the ECG such as the P wave due to their clinical relevance.

Alternative algorithms to increase robustness such as the use of committees or the implementation of genetic algorithms to define the initial weights in the ANN will be explored. We will also consider approaches to overcome the lack of explainability of the ANN approach.

As mentioned before, and supported by [9], using general coefficients to perform the reconstruction would lead to less accurate results. We suggest that, once the specific coefficients are obtained, they can be analyzed to infer some possible relationship between patients with similar conditions or physiognomies that would allow to speed up the training of the models, which would not necessarily start from scratch, applying some kind of transfer learning to these models [38].

These assumptions must be re-evaluated with the existence of new data, if possible pathological, to confirm, on the one hand, their diagnostic effectiveness and, on the other hand, whether the hypotheses of this work are correct. In addition, certain relationships between pathologies or patient physiognomies may appear to be of interest.

## Figures and Tables

**Table 1:** *p*-values of the independence test between the control reconstruction algorithm, the Linear Regression, and the rest of the ECG reconstruction algorithms.

	P-Wave Seg.	ANN/Lead	All-Lead ANN
RMS	0.2144	<0.001	<0.001
CC	0.2626	<0.001	<0.001
MAD	0.7928	<0.001	<0.001
SSD	0.2220	<0.001	<0.001
SNR	0.2821	<0.001	<0.001

**Table 2:** Results obtained in the reconstruction showed in Figure 8 for the proposed location. The average values of each FoM for the 12 leads are shown.

RMS(μV)	CC(%)	MAD(μV)	$\mathrm{SSD}({\mathrm{mV}}^{2})$	SNR(dB)
12.99	99.73	70.19	18.46	22.61
